# Chitosan-Recombinamer Layer-by-Layer Coatings for Multifunctional Implants

**DOI:** 10.3390/ijms18020369

**Published:** 2017-02-09

**Authors:** Jeevan Prasaad Govindharajulu, Xi Chen, Yuping Li, Jose Carlos Rodriguez-Cabello, Mrinal Battacharya, Conrado Aparicio

**Affiliations:** 1 Department of Bioproducts and Biosystems Engineering, University of Minnesota, St. Paul, MN 55108, USA; gprasaad88@gmail.com; 2 Department of Restorative Sciences, School of Dentistry, University of Minnesota, Minneapolis, MN 55455, USA; chenx838@umn.edu (X.C.); lixx1191@umn.edu (Y.L.); 3 BIOFORGE Group, University of Valladolid, Valladolid 4701, Spain; roca@bioforge.uva.es

**Keywords:** chitosan, elastin-like recombinamers, layer-by-layer, titanium, implant

## Abstract

The main clinical problems for dental implants are (1) formation of biofilm around the implant—a condition known as peri-implantitis and (2) inadequate bone formation around the implant—lack of osseointegration. Therefore, developing an implant to overcome these problems is of significant interest to the dental community. Chitosan has been reported to have good biocompatibility and anti-bacterial activity. An osseo-inductive recombinant elastin-like biopolymer (P-HAP), that contains a peptide derived from the protein statherin, has been reported to induce biomineralization and osteoblast differentiation. In this study, chitosan/P-HAP bi-layers were built on a titanium surface using a layer-by-layer (LbL) assembly technique. The difference in the water contact angle between consecutive layers, the representative peaks in diffuse reflectance infrared Fourier transform spectroscopy (DRIFTS), X-ray photoelectron spectroscopy (XPS), and the changes in the topography between surfaces with a different number of bi-layers observed using atomic force microscopy (AFM), all indicated the successful establishment of chitosan/P-HAP LbL assembly on the titanium surface. The LbL-modified surfaces showed increased biomineralization, an appropriate mouse pre-osteoblastic cell response, and significant anti-bacterial activity against *Streptococcus gordonii*, a primary colonizer of tissues in the oral environment.

## 1. Introduction

Orthopedic implants can restore the patient’s physical condition, thereby improving their quality of life. There are several types of implants, including hip, knee, spine, maxillo-facial, and dental implants. The two most relevant contemporary problems facing orthopedic and dental implants are infection and the lack of rapid implant integration into the adjacent bone tissue (osseointegration). These issues are particularly notable for dental implants, and are primarily responsible for most of their failures [1,2]. A bacterial biofilm surrounding the surface of the implant leads to inflammation. The diseases that are associated with this inflammation are termed peri-implant diseases, and are of two types: peri-implant mucositis and peri-implantitis.

To date, commercially-pure titanium has been extensively used to make dental implants [1], because of its suitable mechanical properties (high tensile strength and elastic modulus), corrosion resistance, and biocompatibility [3]. However, titanium lacks the ability to rapidly osseointegrate. Biomolecules such as the oligopeptide arginine-lysine-aspartate (RGD), have been immobilized on the implant surface to attract the osteoblast cells, by inducing specific interactions with the cellular integrin receptors [1]. Poly-l-lysine (PLL) has been electrostatically coupled with the titanium oxide layer, which in turn aided in RGD immobilization [4]. Other techniques that promote osseointegration include coating implants with peptide aptamers [5] or recombinant fibronectin (FN)-derived oligopeptide [6], and modifying surfaces using heparin chemistry [7].

Calcium phosphate (CaP) coatings, which are stoichiometrically similar to hydroxyapatite (HAP), can improve osseointegration [3]. Mimicking the natural chemistry of the mineral in bone HAP induces beneficial responses in the biological environment for osteoblast cells, allowing them to attach, proliferate, and differentiate [8,9]. While CaP coatings on the implant surface may increase the rate of osseointegration, there is still the issue of bacterial colony-formation [10].

The application of proteomics in dentistry has enabled important milestones to be achievedover the last 15 years [11]. The study of the proteome of saliva and crevicular fluid has aided in discerning the bioactivity of a number of biomolecular components (proteins and peptides) present in the oral environment. More than 40 antimicrobial peptides and proteins are expressed in the oral cavity [12,13], and other proteins are known to have an active role in controlling the biomineralization processes on teeth. Statherin is a 43-residue multifunctional protein that is present in human saliva, and which can regulate the mineralization occurring in the oral cavity and tissues [14]. This protein has a strong affinity to attach to enamel, which is composed almost entirely by HAP [15]. The 15 amino acid N-terminal of statherin, the so-called SN15 peptide (DSpSpEEKFLRRIGRFG), has been reported [16] to be responsible for the protein’s high affinity to HAP. The high negative-charge of this N terminal region and the helical conformation, are primarily responsible for its preferential adsorption on HAP [17]. The SNA15 sequence (DDDEEKFLRRIGRFG) has an affinity with HAP that is comparable to that of native SN15. So, this sequence can be used to form HAP [16], which can aid in bone formation [18,19]. Elastin-like recombinamers (ELRs) are a class of recombinant polypeptides which recapitulate the physical and chemical properties of native elastin [20], such as the elasticity, and the reverse transition with temperature. The SNA15 sequence has been incorporated in the recombinant polymers of elastin, and these polymers have shown the ability to induce biomineralization [21,22,23].

The bacterial colonization of titanium eventually leads to implant-failure [24]. This is because bacterial colonization leads to a biofilm formation that is believed to aid the bacteria in evading the host defense mechanism and antibiotics [25]. Pathogens eventually cause bone loss around the implant, resulting in the necessity for surgery to be performed, either to remove the affected bone and/or to replace the infected implant. The infected site is then treated with systemic antibiotics to eradicate the presence of bacteria. Hence, there is a need to incorporate anti-microbial properties into the dental implant.

Fluorine and silver ions are known for their anti-bacterial activity, as they can bind themselves to proteins present inside the bacteria, inhibiting cellular activities [26,27]. These ions have been incorporated on the surface of titanium and have proved to be effective against bacterial biofilm formation [27]. Antibiotics-releasing coatings are effective only in the short run, which implies that this approach cannot prevent peri-implantitis after several years of implantation [1]. Coatings made of immobilized antimicrobial oligopeptides have also been used [28,29]. The use of polymeric materials with antimicrobial properties are another option. Antimicrobial properties are inherent in their original structure, as a result of chemical modification, or by the introduction of organic or inorganic antimicrobial agents [30,31].

Chitosan is known for its biocompatibility and anti-bacterial activity against a number of pathogens [32,33,34]. The mechanism for the anti-bacterial activity of chitosan remains unclear, but it is believed that the positively-charged amines in chitosan attract the negatively-charged bacterial cell wall, causing it to disturb the cell dynamics and/or disruption of the cell membrane [33,35,36]. The number of amino groups present in chitosan is believed to influence its ability to kill the bacteria [36]. Since the anti-bacterial activity of chitosan is contact-dependent, the bacteria can grow away from the chitosan membrane [35]. Apart from being an anti-bacterial agent, chitosan is also reported to be an excellent material for growing osteoblast cells [37,38,39], because it has structural characteristics that are similar to various glycosaminoglycans and hyaluronic acid [40].

The objective of this work is to develop a coating that will be multifunctional; i.e., effective in inducing biomineralization and fast bone growth, as well as preventing peri-implant infections. The positioning of P-HAP/Chitosan coatings on a commercially pure titanium surface, using the Layer-by-Layer (LBL) assembly technique, have been developed to obtain a coating that will induce positive responses on osteoblast cells and kill the bacteria associated with the formation of biofilms in the oral environment. The positively-charged chitosan (at acidic pH) and the highly negatively-charged P-HAP can effectively form bi-layers, due to electrostatic attraction.

## 2. Results and Discussion

### 2.1. Layer-by-Layer Assembly

**Figure 1:**
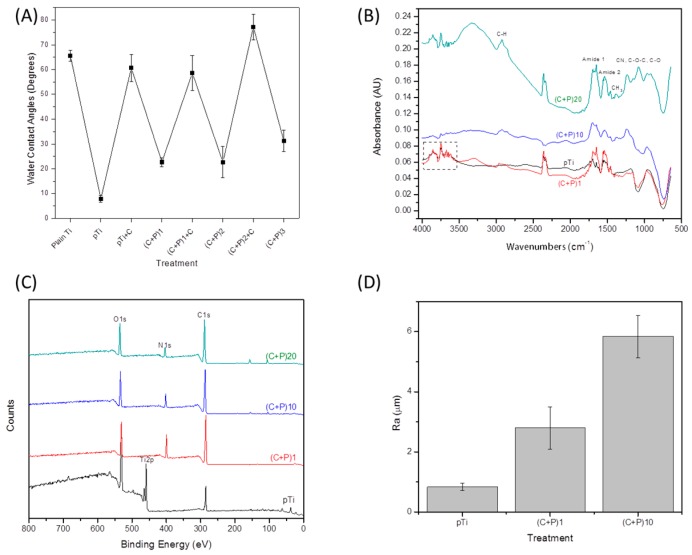
Physical and Chemical Properties of the layer-by-layer (LbL) coatings. (**A**) Water contact angles up to three bi-layers; (**B**) Diffusive reflectance Fourier-transfor infrared spectroscopy (DRIFTS) spectra; (**C**) X-ray photoelectron spectroscopy (XPS) surveys; and (**D**) values of average roughness (*R*_a_) calculated from Atomic Force Microscopy (AFM) imaging of Ti surfaces (pTi or plasma) and Ti coated with one bilayer ((C + P)_1_), 10 bi-layers ((C + P)_10_), and 20 bi-layers ((C + P)_20_).

The contact angle of the titanium surface was expected to change with the multi-layer buildup, due to the change in the hydrophilicity of the surface. The water contact angle of the untreated (plain) titanium surface was approximately 65° (Figure 1A). After plasma cleaning, a significant 8° drop in the contact angle was observed, as plasma cleaning removes any contaminants on the surface, and thus, polar hydroxyl groups are readily available to interact at the surface/water interface, producing highly hydrophilic surfaces. After the adsorption of chitosan on the surface, the contact angle increased to approximately 60°, in accordance with previously reported contact angle values for the chitosan adsorbed on titanium surfaces [39,41,42]. After the P-HAP been adsorbed, following the chitosan adsorption, there was a significant decrease (*p* < 0.05) in the water contact angle, to a value of approximately 22°. The difference in the contact angle between each consecutive treatment (i.e., between pTi and Ti + C, Ti + C and (C + P)1, and so on) was statistically significant (*p* < 0.05). There was no significant difference between the (C + P)1 and the (C +P )2 groups. Similarly, no significant difference was observed between the Ti + C and the (C + P)1C groups. This indicates that only the outermost layer on the surface changed the value of the contact angle, as the layers were added. Also, the contact angle values did not change significantly (*p* > 0.05) when the same polymer as their outermost layer was present (like Ti + C and (C + P)1C). This “zigzag” behavior of the contact angle during LbL assembly has been often reported [36,41,42,43,44], and validates the proper multi-layer buildup.

The LbL assembly of chitosan and the protein (P-HAP) on titanium was carried out at pH 4.5, which provided a negatively-charged titanium surface and positively-charged cationic chitosan molecules. Thus, electrostatically-attracted chitosan formed the first stabilized layer on the titanium. The samples were then treated with the P-HAP solution, which was anionic, at pH 4.5. The electrostatic attraction between the chitosan and the P-HAP drove the formation of a bi-layer. After each layer’s adsorption, the samples were washed in water to remove any of the loosely adhered molecules. The samples were air-dried to remove the water molecules (which were adsorbed during the wash routine). The removal of the water molecules was important, since both of the molecules involved (chitosan and P-HAP) were hydrophilic and could attract a layer of water molecules on their surface, hindering the multi-layer buildup. The water used for washing was also maintained at pH 4.5, so that the charge of the adsorbed molecules did not change the electrostatic force of attraction.

The DRIFTS spectra were used to confirm the presence of chitosan and P-HAP in the LbL coatings. The spectrum of the one bi-layered sample was no different from that of the plasma-treated titanium sample, attributed to the fact that the penetration depth of DRIFTS is in the order of a few micrometers, while that in the LbL assembly would be in the range of a few nanometers [45,46]. As a result, visible spectral peaks for chitosan and the P-HAP were only observed upon the addition of several layers (10 and 20 bi-layers) over the titanium surface.

The broad peak, at around 3300 cm^−1^ (Figure 1B), can be assigned to the O–H groups present in the chitosan molecules and the N–H stretches of both chitosan and P-HAP [47,48,49]. In addition, this broad peak can also be assigned to the O–H stretching of water [50]. Peaks in the region of 2900 cm^−1^ may be attributed to the C–H stretch [47,49], which arises from both the chitosan and the P-HAP molecules. The bands from 1650 to 1700 cm^−1^ may be attributed to the amide I vibration, which was generated due to the C=O stretching vibration, which is present in both the chitosan and the P-HAP molecules [47,48]. The amide II band, at 1530 cm^−1^ [47,49], can be noticed in the spectra, which is due to the NH bending vibration, present in both of the molecules. It is noted that the amide I and amide II bands can also be observed on the plasma-treated titanium surface, but the peaks collected for the multilayered surface, especially the 20 bi-layered surfaces, are stronger. The CH_3_ symmetric deformation, which is characteristic of chitosan, can be assigned to the peak shown in Figure 1B, at approximately 1380 cm^−1^ [47,49]. There are three peaks between 1070 to 1250 cm^−1^, which can be attributed to the C–N stretch, present in chitosan [47] and P-HAP, the C–O–C stretch present in chitosan, and the C–O stretching vibration which is also present in chitosan [47,49].

**Figure 2:**
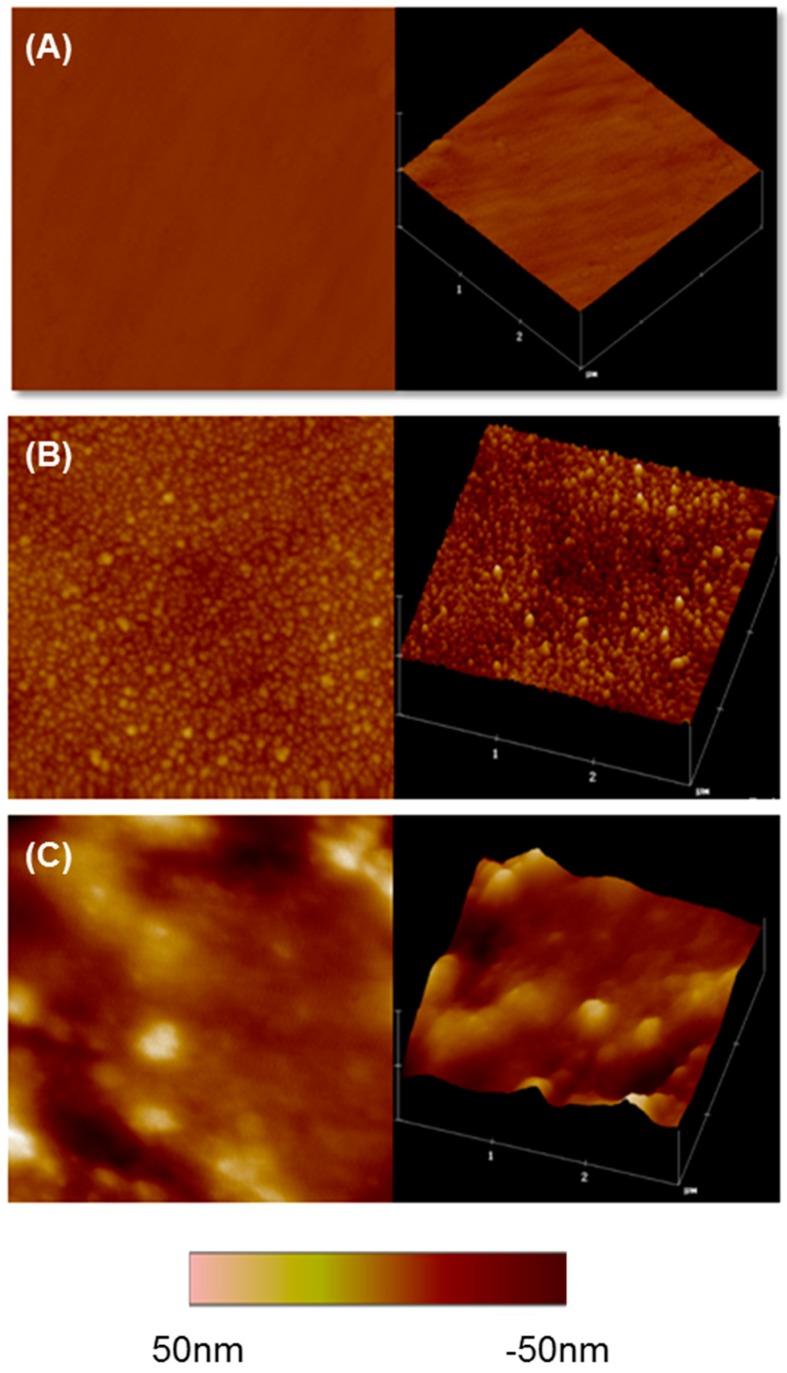
AFM images of non-coated and LbL-coated surfaces. (**A**) pTi non-coated surface; (**B**) One bi-layered Ti surface; and (**C**) ten bi-layered surface. Two-dimensional (**left**) and three-dimensional (**right**) representative images of the surfaces are shown. Scanned area shown is 3 × 3 µm.

The topographies of the chitosan/P-HAP LbL-modified surfaces were analyzed using the tapping mode AFM [51]. The uncoated plasma-treated titanium surface displayed a smooth topography, whereas the LbL-modified surfaces with one and 10 bi-layers showed granular structures, which were evident from the two-dimensional and three-dimensional images (Figure 2). The size of these granules increased with the number of bi-layers. Granules on the ten bi-layer surface were considerably larger than those on the one bi-layered coating. This kind of granular growth in LbL assembly has been previously reported [52]. The average roughness (*R*_a_) increased as the number of bi-layers increased (Figure 2A–C). The granular structures present in the LbL-modified surfaces can be attributed to the characteristic behavior of the P-HAP molecule; i.e., when above the reverse transition temperature, clear nano-sized agglomerates are formed, which may result from the collapse of adjacent P-HAP chains in the surface. Similar observations involving the LbL assembly of P-HAP and chitosan on a glass surface have been reported [21].

The elemental composition of the surface was determined using XPS. The nitrogen-containing groups are present in both chitosan and P-HAP, but are absent in the plasma-treated titanium surface (Figure 2C). These nitrogen-containing groups, which are specific to chitosan and P-HAP, were used as indicators to confirm the deposition of bi-layers on the surface. It is important to note that the titanium peak disappeared in all LbL-modified surfaces, even in the case of one bi-layer. This indicates that the surface was completely covered by chitosan and P-HAP molecules, starting from the very first bi-layer. Given the penetration depth of XPS and the area from which the electrons were extracted (from around 5 nm depth [53]), it may be inferred that each bi-layer was of at least a minimum 5 nm thickness.

### 2.2. Biomineralization

**Figure 3:**
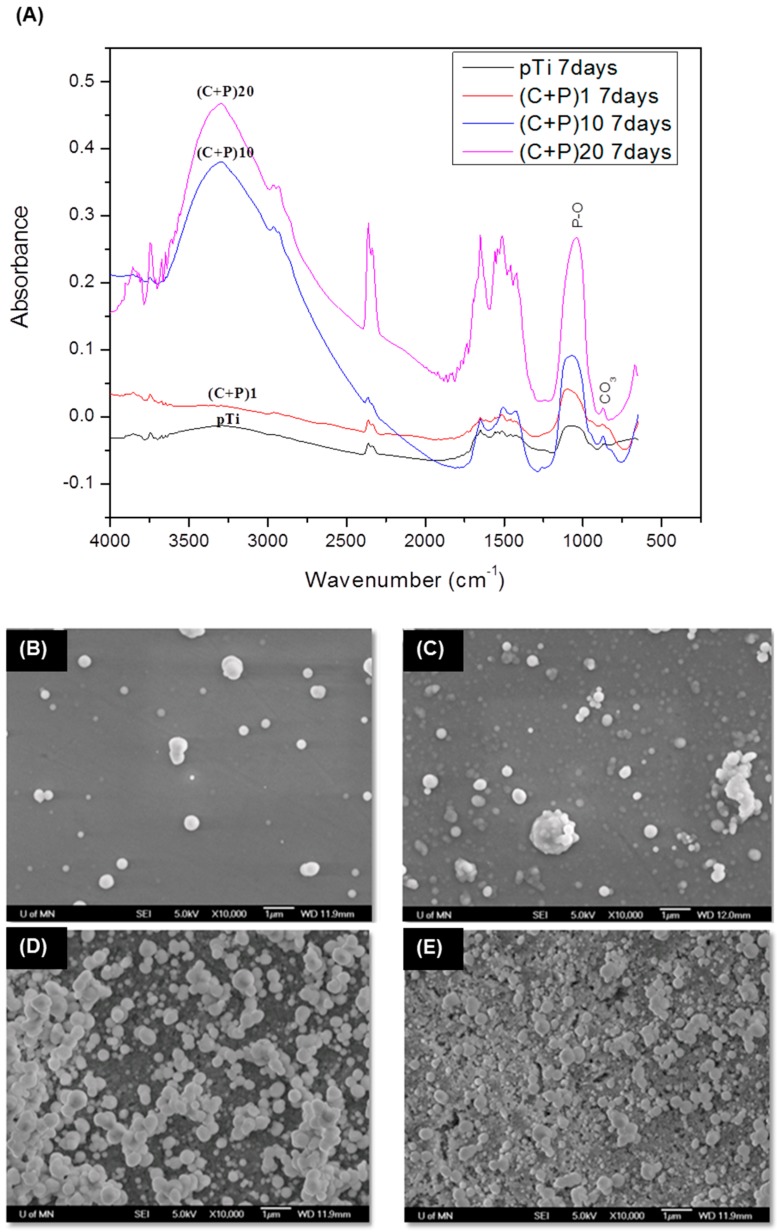
Biomineralization of LbL surfaces. (**A**) DRIFTS spectra of the biomineralized pTi and LbL-coated surfaces; (**B**–**E**) SEM pictures of biomineralized surfaces (**B**) non-coated pTi surface; (**C**) One bi-layered ((C + P)_1_); (**D**) 10 Bi-layered ((C + P)_10_); and (**E**) 20 bi-layered surfaces ((C + P)_20_).

The ability of the LbL-modified and unmodified (pTi) titanium samples to induce calcium phosphate mineralization was analyzed. The DRIFTS spectra of the biomineralized samples are shown in Figure 3A. Amide I and II peaks were visible in the biomineralized samples. The peak, at around 1380 cm^−1^ (CH_3_ symmetric deformation), is hardly noticeable in the spectra for the biomineralized samples, attributed to the formation of a thick calcium phosphate mineral layer on the surface. There is a broad peak from 900 to 1170 cm^−1^, corresponding to the biomineralized samples, due to the overlapping of the P-O symmetric and anti-symmetric stretching vibrations [50,51,54,55,56,57] of the PO_4_^3−^ groups. The intensity of the DRIFTS signal substantially increased as the number of bi-layers also increased, which is an indication that the amount of minerals increased with the increased number of bi-layers. The peak, at 863 cm^−1^, was attributed to the carbonate group present in carbonated calcium phosphate minerals, which has been previously reported for in vitro biomineralization processes [55,58]. Although there are other peaks (amide I and II) which can be attributed to the P-HAP in the spectra of biomineralized samples, the presence of several proteins in the biomineralized layer from osteogenic media cannot be ignored. The almost complete disappearance of the chitosan-specific CH_3_ peak suggests that the minerals thoroughly covered the LbL-coated surface. The latter was confirmed by SEM visualization of the mineralized surfaces after seven days of incubation in osteogenic media (Figure 3B–E). All of the surfaces showed minerals on them. When a biomaterial is immersed in SBF, the continual deposition of the calcium phosphate layer on the surface occurs by precipitation, and the growth of calcium phosphate nanocrystals agglomerates [59]. Similar results were observed in this study.

The highest amount of mineral accumulation was observed for (C + P)_20_, followed by (C + P)_10_, (C + P)_1_, and pTi, respectively. The reason for the increased amount of mineralization could be attributed to the increased amount of P-HAP as the number of bi-layers increased, which attracted a higher number of calcium and phosphate ions to these surfaces. It was noted that the minerals on the one bi-layered surface ((C + P)_1_), were growing on a previously developed and homogeneously formed mineral layer. This pattern was also observed on other LbL-modified surfaces (10 and 20 bi-layers). The calcium-to-phosphate molar ratio (Ca/P), calculated for the different biomineralized layers, increased from 1.56 for surfaces with one bi-layer, to 1.62 for surfaces with 20 bi-layers. The resulting Ca/P ratios were close to the values for neonatal bone [60].

### 2.3. Osteoblast Response

**Figure 4:**
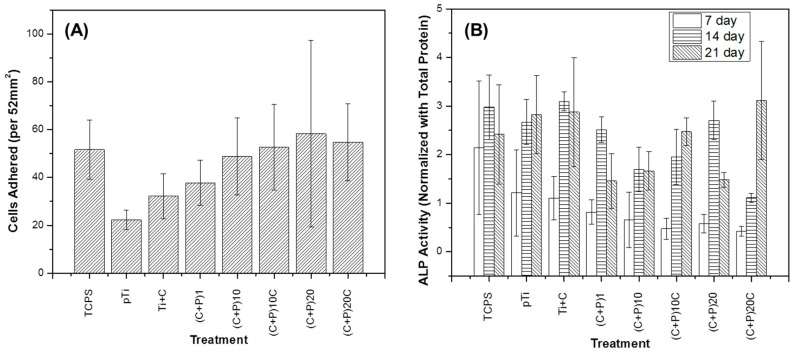
Osteoblast response on LbL surfaces. MC3T3 (**A**) adhesion after 4 h and (**B**) alkaline phosphatase activity after seven, 14, and 21 days of culture on non-coated pTi surface; (**B**) one bi-layered ((C + P)_1_), 10 bi-layered ((C + P)_10_), 20 bi-layered surfaces ((C + P)_20_), and an additional layer of chitosan for the different layered surfaces (Ti + C, (C + P)_10_C, and (C + P)_20_C).

There were no statistically significant changes in the number of adhered cells on Ti surfaces with different LbL coatings (Figure 4A); however, as the number of bi-layers increased, the number of adhered cells also increased, to the values of the positive control (TCPS). The increased number of adhered cells on the surface is an indication that the LbL-modified surfaces are cytocompatible, and that increasing the number of signaling molecules, benefits cell adhesion.

Alkaline Phosphatase (ALP) activity is an early osteoblast differentiation marker. As seen in Figure 4B, LbL modification on the titanium surface did not induce increases in the ALP activity, as hypothesized. In fact, after 14 days of culture, the ALP activity was seen to be lower in coated samples than the controls, except for the titanium surfaces with the (C + P)_2__0_ treatment. The (C + P)_2__0_C and the (C + P)_1__0_ treatments were significantly lower (*p* < 0.05) than the positive TCPS control.

Overall, the cellular experiments suggest that the LbL coatings did not have a significant effect on the osteoblastic adhesion and differentiation. This response may be due to an antagonistic effect between the increased biological signals provided by the biomolecules, and the detrimental decrease in the stiffness of the substrate. Previous reports have concluded that a decrease in surface stiffness compromises the differentiation of cells towards the osteoblastic lineage [61]. Thus, further studies should be conducted to obtain stiffer LbL layers, either by cross-linking the layers or combining them with nanofibers of biocompatible materials.

### 2.4. Antibacterial Activity

**Figure 5:**
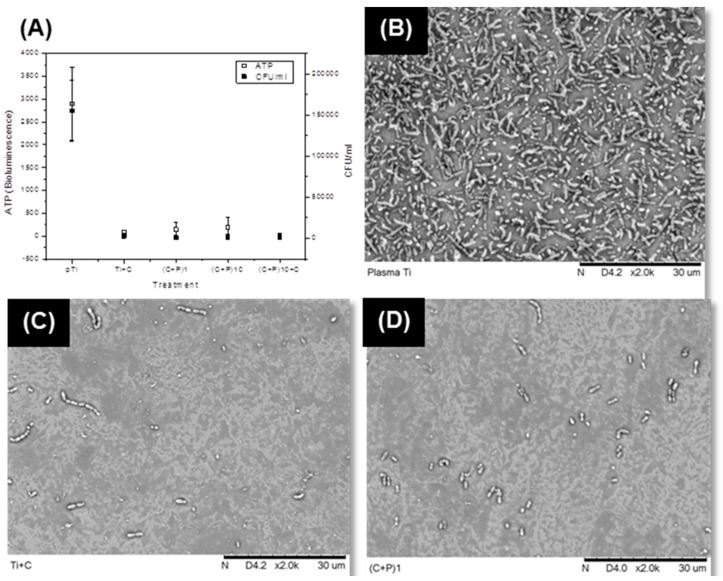

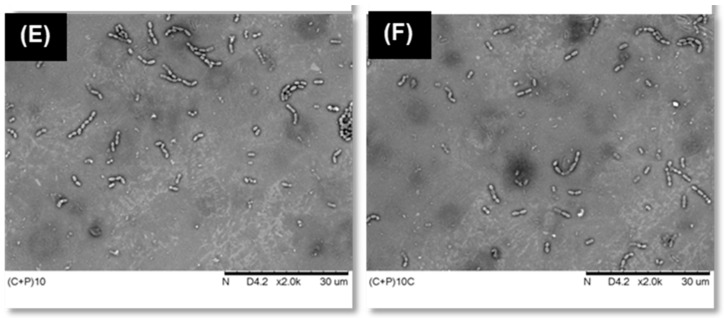
Antibacterial acivity of LbL surfaces against *S. gordonii*. (**A**) ATP and CFU of *S. gordonii* bacteria cultured on non-coated pTi and different LbL-coated surfaces (**B**–**D**) SEM pictures of *S. gordonii* bacteria on (**B**) pTi; (**C**) pTi with one layer of chitosan (Ti + C); (**D**) One bi-layered ((C + P)_1_); (**E**) 10 bi-layered ((C + P)_10_); and (**E**) 10 bi-layered surface with one additional chitosan coat as the outermost layer ((C + P)_10_C).

The gram-positive *Streptococcus gordonii*’s response to the LbL-modified surfaces was also analyzed. *S. gordonii* has been found in the microbiota of bacterial colonization immediately after installation of oral implants, as well as on locations associated with dental peri-implantitis. Therefore, strategies that prevent *S. gordonii* adhesion on the surface compromise the biofilm formation, and therefore can minimize the risk of developing peri-implantitis [29]. Colony forming units (CFU) and ATP activity of the bacteria were significantly reduced in all of the LbL-modified surfaces (Figure 5A). Even the surface with one layer of chitosan (Ti + C) showed a significant reduction, demonstrating the anti-bacterial efficacy of the chitosan layer. The number of viable bacteria, i.e., CFU/ml was reduced to at least 50-fold, in the case of LbL-modified surfaces. The CFU/mL of the LbL-modified surfaces (Ti + C, (C + P)_1_, (C + P)_10_, and (C + P)_10_C) was found to be significantly lower (*p* < 0.05) than that of pTi. Since the ATP and CFU data were highly correlated (*r* = 0.99), we can conclude that the low metabolic activity (ATP) in LbL-modified surfaces was due to a reduction of viable bacteria on the tested surfaces, which resulted in lower CFU/mL. The number of amino groups present in chitosan is believed to influence its ability to kill the bacteria [36]. Another suggested mechanism is the formation of polyelectrolyte complexes, as a result of the interaction between the chitosan and the bacterial cell wall [62,63], due to which, the nutrient permeation ceases. Chitosan treatment has caused a significant change in the expression of many genes in bacteria. These results indicate that the mode of action is complex and that there cannot be a single pathway by which chitosan can kill the bacteria. Hence, it is expected that the number of amine groups is higher with an increased number of bi-layers, which can ultimately enhance the anti-bacterial activity. In this study, however, the anti-bacterial activity was independent of the number of bi-layers. This suggests that a notable number of positively-charged amine groups were consumed for ionic bonding with the acidic groups in P-HAP, during LbL assembly. Antimicrobial properties have been reported for the C-terminal peptide of statherin; however, SN_A_15, the statherin peptide incorporated in P-HAP, is the N-terminal peptide of this protein.

The SEM pictures (Figure 5B–F) show that bacterial adhesion was reduced, as a result of LbL modification. To test P-HAP specific antimicrobial activity, P-HAP was adsorbed on plasma-treated samples and tested against bacteria colonization. There were no notable differences in the number of bacteria adhered on the P-HAP coated and non-coated control surfaces, which suggested that the anti-bacterial effect caused by LbL-modified surfaces was primarily due to the presence of the chitosan molecules in the coatings. Generally, it is believed that the chitosan acts as an anti-bacterial agent by attracting the bacteria towards it and disturbing the cell wall dynamics [33,35,36].

*S. gordonii* is a primary colonizer on oral tissues and synthetic substrates in the oral cavity, that enables adhesion for the pathogenic biofilm formation by *P. gingivalis* [64]. If *S. gordonii* cannot attach to the surface, *P. gingivalis* is unable to notably attach to it and thus, the pathogen becomes more susceptible to being removed from the potentially exposed surface [65].

## 3. Materials and Methods

All of the chemicals were purchased from Sigma-Aldrich (Saint Louis, MO, USA), unless indicated. The mouse pre-osteoblastic cell line MC3T3-E1 subclone 15 was provided by Eric Jensen, School of Dentistry, University of Minnesota (Minneapolis, MN, USA). The *Streptococcus gordonii* (ML-5) strain was provided by Helmut Hirt, Diagnostic and Biological Sciences, University of Minnesota (Minneapolis, MN, USA). Statherin-derived bio-polymer (denoted as P-HAP), with an amino acid sequence ([(VPGIG)_2_ (VPGKG) (VPGIG)_2_]_2_ DDDEEKFLRRIGRFG [(VPGIG)_2_ (VPGKG) (VPGIG)_2_]_2_), was fabricated as previously reported [21,66,67]. Titanium discs (6 mm diameter) were cut from commercially pure grade II titanium (McMaster Carr, Elmhurst, IL, USA).

### 3.1. Sample Preparation

The commercially pure titanium (Ti) discs were polished using 240 and 600 grit silicon carbide discs (Buehler, Lake Bluff, IL, USA), for five minutes. This was followed by further polishing using 1 µm and 0.5 µm alumina suspension (Buehler), for one hour. The polished samples were soaked overnight in acetone solution and washed with de-ionized water. The discs were then ultra-sonicated in cyclohexane (BDH Chemicals, West Chester, PA, USA) solution for 15 min, and washed thoroughly with acetone and de-ionized water. The discs were then dried in a flow of nitrogen.

### 3.2. Fabrication of Layer-by-Layer Assembly

The layer-by-layer technique involves the electrostatic assembly of positively- and negatively-charged polyionic compounds on solid substrates, through van der Waals interactions. The chitosan solution (1 mg/mL) was prepared by dissolving chitosan (coarsely ground flakes and powder, deacetylation ≥75%, Molecular weight >310,000 g/mol) in 2% (*v*/*v*) acetic acid solution (Ricca Chemical Company, Arlington, TX, USA). The P-HAP (molecular weight of 32 kDa) solution (0.5 mg/mL) was prepared in cold water (4 °C). The pH of both of the solutions was adjusted to 4.5, using hydrochloric acid and sodium hydroxide. At this pH, the chitosan was positively-charged, whereas the biopolymer and titanium dioxide were negatively-charged. The polished Ti discs were cleaned using O_2_-Plasma Cleaner PDC-32G (Harrick Plasma, Ithaca, NY, USA), for 5 min. The plasma-treated Ti discs (pTi) were immersed in chitosan solution for 30 min. The samples were then rinsed using de-ionized water (pH 4.5), and dried. Next, the samples were immersed in P-HAP solution for 30 min, rinsed using de-ionized water, and dried. This cycle was repeated to obtain the desired number of bi-layers.

### 3.3. Characterization of Coated Surface

The contact angle for pure titanium and after each layer build-up, was measured using the sessile drop method (using 3 µL of de-ionized water). The drop was equilibrated on the surface for 30 s and the contact angle measurement was recorded for every second, using a DM-CE 1 Contact Angle Meter (Kyowa Interface Science, Saitama, Japan). Since it took approximately 25 s for the droplet to stabilize, the last four measurements (values of 27th–30th second) were averaged for each sample. Three samples were used per treatment.

Diffuse reflectance Fourier transform infrared spectroscopy (DRIFTS) (Nicolet Instruments, Madison, WI, USA) was used to determine the characteristic peaks of chitosan and P-HAP. The wavenumber scan ranged from 648 to 4000 cm^−1^. Plasma-treated Ti discs were used as control backgrounds to ascertain the characteristic peaks of the LbL-modified surfaces.

The AFM characterization was done using a Dimension 3000 AFM (Digital Instruments, Santa Barbara, CA, USA) in tapping mode at room temperature, to monitor the topography of the LbL-modified surfaces. The Average Roughness (*R*_a_) and Surface Area Difference (SAD) in % were determined for each image. Two-dimensional and three-dimensional images were taken to better understand the surface topography. Four different areas were scanned for each sample.

The elemental chemical composition of the LbL-modified surfaces was obtained using X-ray Photon Spectroscopy (SSX-100, Surface Science Instruments, Mountain View, CA, USA), with a focused monochromatic Al Kα X-ray source. The electron take-off angle was 350°, with a spot size of 800 µm. The scan range was from 0 to 1100 eV. One sample per treatment was analyzed.

### 3.4. Biomineralization

The multilayered samples were mineralized with an enzyme-directed biomimetic mineralization process [28], placed in a 48 well plate and incubated in 1 mL of MEM-α medium, supplemented with 20 mM CaCl_2_·2H_2_O, 10 mM β-glycerol phosphate, 50 µg/mL ascorbic acid, 10% HyClone™ fetal bovine serum, and 1% penicillin-streptomycin, at 37 °C for seven days. The medium was replaced every three days. Following the incubation period, the samples were dehydrated using increasing concentrations of ethanol, from 60% to 100%. Plasma-cleaned samples were used as controls for this experiment.

### 3.5. Characterization of Biomineralization

The biomineralized samples were analyzed using DRIFTS to find the characteristic peaks of phosphate. Plasma-treated Ti discs were used as the background. The samples were viewed using a tabletop Scanning Electron Microscope (TM 3000, Hitachi, Tokyo, Japan), to visualize calcium phosphate mineral deposition on the multilayered samples. Images were taken at 10,000× and 20,000× for each sample. The images were obtained as combinations of the collated secondary and back-scattered electrons and thus, the images carried topographical and elemental/chemical information. To analyze the Calcium-to-Phosphorus (Ca/P) ratio, an X-ray energy dispersive spectroscopy EDS microprobe with Quantax 70 software (Bruker, Fitchburg, WI, USA) was attached to the SEM equipment. Three different areas were analyzed per sample.

### 3.6. Osteoblast Cell Culture

The mouse pre-osteoblast cell line (MC3T3-E1) was maintained in a complete culture medium, consisting of MEM-α supplemented with 10% fetal bovine serum and 1% Penicillin-Streptomycin, under standard cell culture conditions (37 °C in a humidified atmosphere of 5% CO_2_). Cells were passaged (sub-cultured) every three to four days. For passaging, the cells were detached using 2 mL trypsin solution (0.075% trypsin in Hank’s Balance Salt Solution from a petri-dish). The complete medium was added in excess to the detached cells and centrifuged at 1500 rpm for 3 min. The cell pellet was re-suspended in the complete medium and transferred into new petri-dishes.

### 3.7. Osteoblast Adhesion

For the adhesion experiment, the treated titanium discs (*n* = 3) were placed in a 12 well plate and incubated in 70% ethanol under UV for 30 min. Wells without titanium discs, which are Tissue Culture Polystyrene (TCPS), served as positive controls. After incubation, ethanol was removed and the wells were washed thrice with Hank’s Balance Salt Solution (HBSS). A total of 200 µL of 5% Bovine serum Albumin was seeded onto the samples (including control) and incubated for 30 min under UV, to block any non-specific cell adhesion. The processed samples were transferred to fresh 48 well plates. Pre-osteoblast cells were diluted using serum-free media (complete media without FBS) and seeded at a density of 2000 cells/well. The cells were allowed to be attached to the samples by incubating them at 37 °C in a humidified atmosphere of 5% CO_2_ for 4 h. After incubation, the cells were fixed using 4% paraformaldehyde for 20 min. The fixed cells were lysed by incubating in the lysis buffer (1× Phosphate Buffered Saline, 0.3% Triton X-100), for 5 min.

A total of 100 µL of Immunofluorescence (IF) solution (prepared by dissolving 1.5 g Bovine Serum Albumin, 5 mL 10× Phosphate Buffered Saline, 0.5 mL 2M MgCl_2_, and 150 Tween 20, in 50 mL double distilled H_2_O) was added to the sample-containing wells and incubated for 30 min, to block non-specific binding. The nuclei of the cells were stained using 4′,6-diamidino-2-phenylindole, dihydrochloride (DAPI). Nucleus staining was used to count the number of cells adhered onto the sample. DAPI- (Invitrogen, Eugene, OR, USA) diluted 1:1500 was mixed in a 1.5 mL microcentrifuge tube. A total of 30 µL of this mixture was added to the samples and incubated for 1 h in the dark, at 37 °C. Finally, the samples were washed thrice and viewed under a Nikon Eclipse E800 fluorescent microscope. Pictures were taken from three different areas per sample and the cells were counted using Image J 1.45 (NIH, Bethesda, MD, USA).

### 3.8. Osteoblast Differentiation

To analyze osteoblast differentiation, the pre-osteoblast cells were seeded at a density of 20,000 cells/well, on to the 48 well plate containing the samples (*n* = 3). Calculations were made so that each well had 800 µL of cell culture. Initially, a complete medium was used, and after confluence (three days after seeding), the media was changed to the differentiation media. The differentiation media comprised MEM-α medium, supplemented with 10 mM β-glycerol phosphate, 50 µg/mL ascorbic acid, 10% HyClone™ fetal bovine serum, and 1% penicillin-streptomycin. Standard cell culture conditions (37 °C in a humidified atmosphere of 5% CO_2_) were maintained. The media was changed every three days. Discs were taken after seven, 14, and 21 days of incubation.

In the plates, the discs were washed with 1× Phosphate Buffered Saline (PBS) to remove any media. The cells were lysed using 300 µL lysis buffer (1% triton X-100, 0.1mM MgCl_2_, and 150 mM tris (Promega, Madison, WI, USA)), at pH 10.5 for 10 min. Then, the cells were manually scraped from the discs into the buffer, using a pipette tip. The scraped cells, along with the lysis buffer, were then transferred into 1.5 mL microcentrifuge tubes and centrifuged at 3000 rpm for 10 min, at 4 °C. The supernatant was collected without disturbing the pellet and stored at −20 °C for alkaline phosphatase activity and total protein quantification.

The measurement of alkaline phosphatase activity involved mixing 5 µL of the cell lysate solution with 100 µL of freshly prepared AMP reaction buffer (22.5 µL of 1:4 diluted 2-amino-2-methyl-1-propanol, 12 µL MgCl_2_, 2 × 20 mg 4-nitrophenyl phosphate disodium salt hexahydrate tablets dissolved in 10 mL ddH_2_O) in each well of the 96 well plate. This mixture was incubated for 2 h at 37 °C and the reaction was stopped by adding 25 µL of 2 M NaOH solution to each well. The absorbance was read at 410 nm using a Beckman Coulter AD-340 spectrophotometer.

The total protein content in the cell lysate was measured in order to normalize the protein differentiation markers with the cell number. A Bio-Rad DC protein assay kit was used to estimate the total protein content. The procedure involved the preparation of protein standards (0.2, 0.4, 0.6, 0.8, 1.0, and 1.2 mg/mL), by diluting bovine serum albumin in lysis buffer. A total of 5 µL of cell lysate solution and protein standards were pipetted into a 96 well plate and mixed with 25 µL of A’ (prepared by mixing 1 mL reagent A and 20 µL reagent S). A total of 200 µL of reagent B was added to this mixture, and the plate was incubated for 15 min. Following incubation, the absorbance was read at 650 nm using a Beckman Coulter AD-340 spectrophotometer. Concentrations of the protein were calculated, based on the standard plot.

### 3.9. Antibacterial Activity

To evaluate the anti-bacterial activity of the samples, a colony of gram-positive *Streptococcus gordonii* was inoculated in 2 mL Bacto Todd-Hewitt broth, and this overnight culture was diluted 10 folds, with 0.9% (*w*/*v*) NaCl solution. This was further diluted 50 folds, with the Todd-Hewitt broth. Titanium discs (*n* = 5) were placed into a 24 well plate, and 1ml of the diluted culture was added to each well and incubated at 37 °C with mild shaking, for 24 h. After the incubation period, the media was taken out and the discs were rinsed with 500 µL NaCl solution, for 5 min. The discs were then transferred to a new 24 well plate and washed thrice for 5 min with NaCl solution, to remove any loosely adhered bacteria on the sides of the discs. After washing, 1 mL of the fresh Todd-Hewitt media was provided to each well containing the discs and incubated for 2 h. The incubation was undertaken to increase the metabolic activity of the bacteria and help them generate more adenosine triphosphate (ATP), so that the sensitivity of the ATP detection could be enhanced. Following the incubation period, the discs were washed thrice with the NaCl solution, as described previously. After a complete washing, four discs per group were transferred into a 1 mL centrifuge tube containing 300 µL NaCl. These tubes were ultra-sonicated for 10 min, to remove the bacteria adhering to the surface. One sample per group was not sonicated, and was used for the SEM analysis.

The ATP quantification was performed using the BacTiter-Glo™ Microbial Cell Viability Assay kit, in order to measure the metabolically active bacteria. The procedure involved mixing 100 µL of the obtained solution after sonication and 100 µL of the BacTiter-Glo™ reagent, in an opaque wall 96 well plate. After 5 min of incubation at 37 °C, the luminescence was measured using a BioTek microplate luminometer.

Colony forming units (CFUs) provide an estimate of the number of viable cells on the surface. Briefly, the procedure involved diluting 100 µL of the obtained solution, serially (10, 100, and 1000 folds). Then, 10 µL of the diluted and undiluted solutions were plated on the Todd-Hewitt Agar plates and incubated overnight at 37 °C, in a humidified atmosphere of 5% CO_2_. The number of CFUs was counted after the incubation period.

In order to know the adhesion pattern of the bacteria on the surface of the coatings, samples were processed and viewed using the scanning electron microscope (SEM). The discs were treated with a primary fixative (2% glutaraldehyde in 0.1 M sodium cacodylate buffer, pH 7.4 with 0.15% alcian blue 8GX) for 1 h at room temperature and then, kept at 4 °C overnight. After the incubation period, the samples were washed with 0.1 M cacodylate buffer for 5 min, and treated with a secondary fixative (1% OsO_4_/0.1 M cacodylate buffer) for 1 h. The samples were then washed with 0.1 M cacodylate buffer for 5 min and dehydrated with increasing ethanol concentrations (50%, 70%, 80%, 95% (twice), and 100% (twice)), for 5 min each. After dehydration, the samples were critical point dried (CPD) with CO_2_ and coated with 50 Å platinum, before being imaged on a tabletop SEM.

## 5. Conclusions

The chitosan/P-HAP bi-layers were successfully assembled on the titanium surface using the Layer-by-Layer technique. The layers were characterized by contact angle measurements, AFM, XPS, and DRIFTS. The DRIFTS spectra, along with the SEM-EDS data, confirmed that the LbL-layers were mineralized with calcium phosphate minerals, using an enzyme-directed biomimetic mineralization process. Also, the amount of mineralization substantially increased with an increased number of bi-layers.

The LbL-modified surfaces were cytocompatible with mouse pre-osteoblast cells and effective against *S. gordonii*, as it showed a 50-fold decrease in the number of viable bacteria (based on colony forming units), when compared to the pTi with no bi-layer. However, the LbL-modified surface did not influence the osteoblast cell differentiation as expected, in spite of its ability to induce biomineralization. This may be due to the lack of rigidity of the obtained biomolecular-based coatings on titanium. Further studies should be conducted to obtain stiffer LbL layers, either by cross-linking the layers or combining them with the nanofibers of biocompatible materials.
